# Efficacy of Ultrasound-Guided Particulate Versus Nonparticulate Steroid Injection in Carpal Tunnel Syndrome: An Open-Label Randomized Control Trial

**DOI:** 10.7759/cureus.21591

**Published:** 2022-01-25

**Authors:** Merrin M Mathew, Ravi Gaur, Nitesh Gonnade, Satyasheel S Asthana, Rambeer Ghuleliya

**Affiliations:** 1 Physical Medicine and Rehabilitation, All India Institute of Medical Sciences, Jodhpur, IND; 2 Physical Medicine and Rehabilitation, Dr. Ram Manohar Lohia Institute of Medical Sciences, Lucknow, IND

**Keywords:** nonparticulate steroid, ultrasound-guided perineural injection, vas, bctq, particulate steroid, triamcinolone, dexamethasone, local corticosteroid injection, cts, carpal tunnel syndrome

## Abstract

Introduction

Perineural corticosteroid injection is an extensively used and accepted treatment for carpal tunnel syndrome (CTS). However, to this date, there is no guideline as to which corticosteroid has to be used as the standard treatment for CTS. Triamcinolone acetonide is a commonly used particulate steroid that can cause permanent nerve injury if it is accidentally injected into the nerve. Conversely, dexamethasone sodium phosphate is a nonparticulate steroid that would not cause permanent nerve damage following accidental injection.

Methods

Mild to moderate cases of CTS, confirmed by nerve conduction studies (NCS), with symptoms greater than three months were recruited. The participants received one session of ultrasound-guided perineural injection by the in-plane axial ulnar-sided approach with 4 mL of either dexamethasone (dexamethasone sodium phosphate 8 mg (2 mL) + 2 mL 0.5% bupivacaine) or triamcinolone (triamcinolone acetonide 40 mg/mL (1 mL) + 2 mL 0.5% bupivacaine + 1 mL normal saline) solution. The parameters assessed were Phalen’s test time (in seconds), visual analog scale (VAS), and Boston carpal tunnel questionnaire (BCTQ) scores at baseline and two and four months, and NCS changes in sensory nerve conduction velocity (SNCV) and distal motor latency (DML) of the median nerve at baseline and four months. Statistical analysis was conducted using the software SPSS version 26.0 (IBM Corporation, Armonk, NY, USA). Independent samples t-test was used for comparison between groups and the paired t-test for improvement within each group. P values < 0.05 were considered statistically significant.

Results

The mean age was 42.64 ± 10.99 in the dexamethasone and 45.22 ± 10.602 in the triamcinolone group cases (P = 0.324).There were 58 females (84.06%) and 11 males (15.94%). Each of Phalen’s test time, VAS, and BCTQ scores significantly improved within both dexamethasone and triamcinolone groups at the second and fourth months after injection (P < 0.05). The NCS parameters (SNCV and DML) also significantly improved in both groups at the fourth month after the injection (P < 0.05). However, there were no significant differences in the improvement of Phalen’s test time between the two groups (P = 0.745), VAS score (P = 0.319), BCTQ score (P = 0.137), SNCV (P = 0.511), or DML (P = 0.753). Postprocedural pain lasted significantly longer in the triamcinolone group (P < 0.05). No major complications were noted in either of the two groups.

Conclusion

Dexamethasone is as effective as triamcinolone in improving the symptoms of CTS and can be used as a safer and more effective alternative in the treatment of mild to moderate CTS cases.

## Introduction

Carpal tunnel syndrome (CTS) is a symptomatic compressive neuropathy of the median nerve at the level of the wrist and accounts for 90% of all peripheral entrapment neuropathies [1]. It is mostly a cumulative trauma disorder (caused by repetitive tasks, forceful exertions, vibrations, mechanical compression, and sustained postures) along with a multitude of other causes such as trauma, inflammatory arthropathy, hypothyroidism, obesity, and diabetes. It affects the day-to-day activities of the affected population, and the associated healthcare costs impose a considerable socioeconomic burden on society, both in terms of productivity loss and treatment costs. The incidence of CTS has been reported to be approximately 276 per 100,000, which is markedly more common in females than in males, and is mostly bilateral with a peak incidence in the age group of 40-60 years [2]. The overall prevalence in the general population was estimated to be approximately 3.8% [3].

CTS is characterized by hand pain, numbness, and tingling sensation in the sensory distribution of the median nerve (lateral 3½ fingers) along with a reduction in grip strength and hand function. Patients commonly complain of a nocturnal worsening of symptoms, worsening of symptoms while driving, inability to hold a handset, gripping issues, and hand clumsiness during daytime with activities requiring wrist flexion. The numbness is aggravated by activities such as typing, driving, or knitting; additionally, nocturnal dysesthesia interrupts sleep and is relieved by shaking or flicking the hand, referred to as the “flick sign” [4].

Diagnosis requires a thorough history of symptom onset, provocative factors, work activity, pain localization and radiation, maneuvers that alleviate the symptoms (Phalen’s test, Tinel’s sign, etc.), and the presence of predisposing factors, such as diabetes, obesity, chronic polyarthritis, myxedema, acromegaly, pregnancy, and sports activities [5]. Phalen’s test has a sensitivity of 85% and a specificity of 90% [6], and Tinel’s test has a sensitivity of 62% and a specificity of 93% [7]. Other tools used for severity assessment include the visual analog scale (VAS), positive Phalen’s test time (in seconds), Boston carpal tunnel questionnaire (BCTQ) developed by Levine et al. in 1993 [8], and others. Nerve conduction studies (NCS) are considered the gold standard for the diagnosis of CTS [9] and require prolongation of motor and sensory latencies in conjunction with reduced sensory and motor conduction velocities of the median nerve [10].

Corticosteroid injection is an extensively used and accepted treatment in mild to moderate CTS according to the guidelines of the American Academy of Orthopedic Surgeons [11] as corticosteroids reduce the inflammation and edema associated with CTS. However, there is no guideline as to which corticosteroid has to be used as the standard treatment in CTS. Triamcinolone acetonide, a commonly used steroid for this indication, is a particulate steroid, which can cause permanent nerve injury if accidentally injected into the nerve [12]. Dexamethasone sodium phosphate is another steroid with a better safety profile that has also been shown to be effective in CTS [12-14]. It is a nonparticulate steroid that would not cause permanent nerve damage even if it is accidentally injected into the nerve [12]. In this study, we compare the efficacy of these two steroids in CTS, which has not yet been studied to date in the Indian population.

## Materials and methods

Aim

In this study, we aim to compare the efficacy of dexamethasone and triamcinolone acetonide injection in patients with CTS.

Objectives

Our primary objective is to compare the positive Phalen’s test time (in seconds) in each group at baseline and two and four months. Our secondary objectives are to compare the pain relief obtained in each group using the VAS at baseline and two and four months, to compare the improvement in the BCTQ score in each group at baseline and two and four months, and to observe the changes in NCS at baseline and four months.

Inclusion criteria

The inclusion criteria included the following: ages between 20 and 80 years and mild to moderate cases of CTS, confirmed by electrophysiological tests, with symptoms lasting for a minimum of three months.

Exclusion criteria

The exclusion criteria were malignancies, cervical radiculopathy, brachial plexopathy, thoracic outlet syndrome, infections, inflammatory joint and connective tissue disorders, uncontrolled diabetes, burns/any local tissue contractures, history of wrist trauma/surgery, and pregnancy.

Sampling and sample size

Dilokhuttakarn et al. (2018) reported an overall positive Phalen’s test time at the two-month follow-up with a mean value of 52.00 with a standard deviation (SD) of 11.42 in the dexamethasone group and a mean value of 42.33 with a standard deviation of 16.95 in the triamcinolone group [15]. Considering this for sample size calculation, we estimated a sample size of 36 in each group with a 95% confidence interval and power of 80%. Thus, a total of 72 patients with CTS were recruited and randomized into two groups.

Methodology and data collection

The study was conducted at the outpatient clinic in the Department of Physical Medicine and Rehabilitation at All India Institute of Medical Sciences (AIIMS), Jodhpur, Rajasthan, India. It was an open-label parallel design randomized control trial conducted from January 2020 to December 2021. Approval was obtained from the scientific committee and Institute Ethics Committee (IEC) of AIIMS Jodhpur prior to the commencement of the study (certificate reference number: AIIMS/IEC/2019-20/968). The entire study was conducted in accordance with the principles of the Declaration of Helsinki, and written informed consent was taken from all the participants prior to the procedure. All patients satisfying the inclusion and exclusion criteria during the study period were considered eligible for participation. The participants were randomized into the dexamethasone and triamcinolone groups using the sequentially numbered opaque sealed envelope technique for allocation concealment, followed by block randomization using block sizes equal to four and five. This was an open-label trial where patients were not blinded to their allocated treatment. All patients clinically diagnosed with CTS and confirmed by electrophysiological studies [10] (using criteria and cutoffs mentioned in Table 1 and Table 2, respectively) received the basic conservative management with hand splint; medications such as gabapentin (300-600 mg), NSAIDs, and paracetamol (up to maximum 4 g/day); and nerve and tendon gliding exercises prior to recruitment for the procedure.

**Table 1:** AANEM grading of CTS based on NCS findings AANEM: American Association of Neuromuscular and Electrodiagnostic Medicine CTS: carpal tunnel syndrome NCS: nerve conduction study SNCV: sensory nerve conduction velocity DML: distal motor latency

CTS grade	NCS finding
Minimal	Abnormal segmental or comparative tests only
Mild	Abnormal SNCV only with normal DML
Moderate	Abnormal SNCV and abnormal DML
Severe	Absent sensory response and abnormal DML
Extreme	Absence of motor and sensory responses

**Table 2:** Abnormal nerve conduction study cutoff values NCS: nerve conduction study SNCV: sensory nerve conduction velocity DML: distal motor latency DSL: distal sensory latency

Nerve conduction study parameters	Abnormal cutoff value
SNCV	<50 m/s
DML	>4.3 ms
DSL	>3.6 ms
Amplitude (sensory)	<10 uV
Amplitude (motor)	<5 mV

The participants in the dexamethasone group received one session of ultrasound-guided perineural injection with 4 mL of dexamethasone solution (dexamethasone sodium phosphate 8 mg/2 mL + 2 mL 0.5% bupivacaine), and those in the triamcinolone group received one session of ultrasound-guided perineural injection with 4 mL of triamcinolone solution (triamcinolone acetonide 40 mg/1 mL + 2 mL 0.5% bupivacaine + 1 mL normal saline). This ensured that nearly equivalent doses of steroids were used in both groups [16]. Then, 1 mL of normal saline was added to the triamcinolone solution to equalize the injectate volumes in both groups while keeping the bupivacaine volume constant. The demographic, clinical, and procedural details of all patients who underwent the procedure, including any adverse reactions, were noted. Ultrasonography was conducted using the Sonosite SII ultrasound machine (FUJIFILM Sonosite, Inc., WA, USA), and NCS was assessed using the Nihon Kohden EMG/EP measuring system (Nihon Kohden Corporation, Shinjuku-ku, Tokyo, Japan). The participants were instructed to refrain from all other CTS treatments throughout the study period.

The entire procedure was conducted under ultrasound guidance following all aseptic precautions in accordance with the standard perineural injection protocol as follows.

Injection Technique

The participants received one session of ultrasound-guided perineural injection by the in-plane axial ulnar-sided approach.

Patient Positioning

The patients were asked to sit with the affected arm resting comfortably on the table. A rolled towel was placed underneath the wrist to create a mild extension.

Probe Position

The transducer was placed along the short axis (transverse) to the median nerve at the wrist. The scan was done proximally and distally until the nerve was clearly identified under the transverse carpal ligament, at approximately the level of the pisiform.

Markings

Because of the shallow needle plane angle, the ulnar nerve and artery were identified, and the needle was inserted just radially or along the depth direction in these structures.

Needle Position

The 1-inch 23 G needle was inserted on the ulnar side of the wrist crease parallel to the transducer for optimal needle visualization using an in-plane approach.

**Figure 1:**
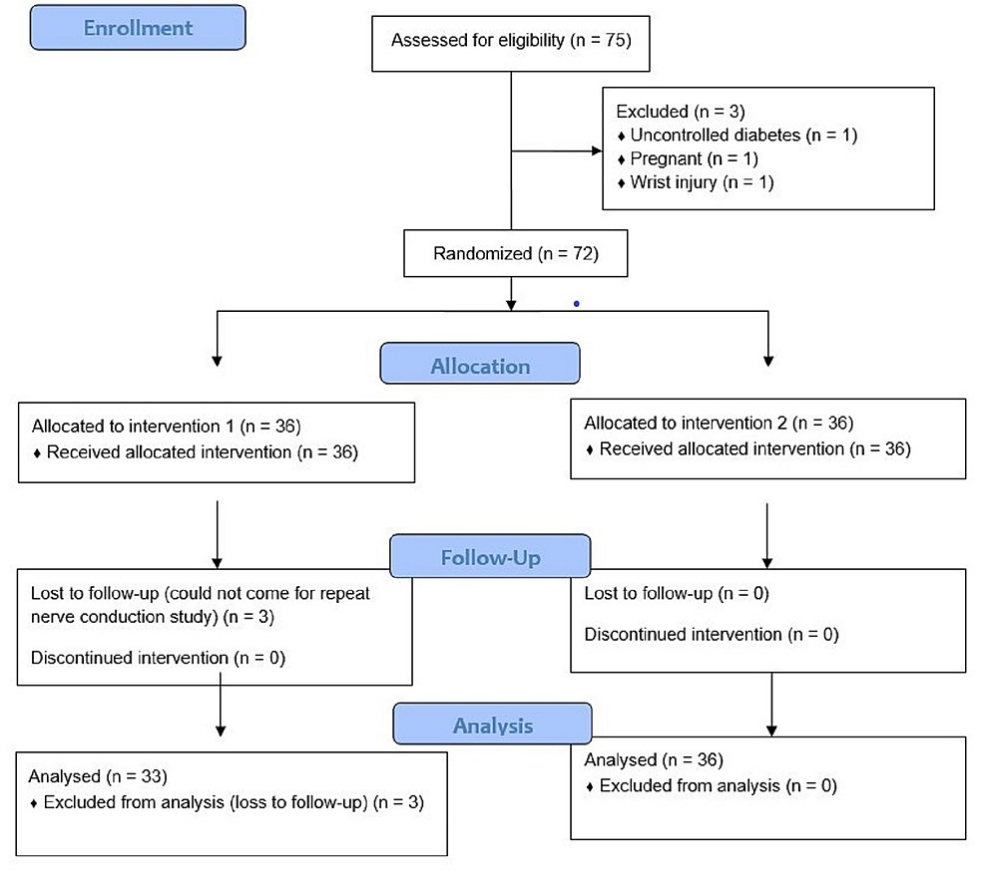
Analysis of the recruited participants

The solution, depending on the randomized grouping of the patient, was injected around the median nerve in the carpal tunnel. After the injection, the participants were advised to apply cold packs at the injection site for three days and to take no other medications for pain apart from paracetamol (up to 4 g/day). Among those who completed the study, 33 patients were in the dexamethasone group and 36 patients were in the triamcinolone group. Figure 1 shows the analysis flowchart of the recruited participants.

The primary outcome was positive Phalen’s test time, and the secondary outcomes included the VAS scored from 0 (no pain) to 10 (unbearable pain), BCTQ score, and NCS changes of the median nerve. The positive Phalen’s test time, VAS, and BCTQ score were evaluated before the treatment (0) and at two and four months after the injection. NCS was evaluated at baseline and four months after the injection.

Statistical analysis

Data were entered in Microsoft Excel (Microsoft, Redmond, WA, USA), and analysis was conducted using SPSS for Windows version 26.0 (IBM Corporation, Armonk, NY, USA). Quantitative variables such as age, positive Phalen’s test time, and VAS score were described using means and standard deviations. Independent samples t-test was used for comparison between groups and paired t-test for improvement within each group. A P < 0.05 was considered statistically significant.

## Results

A total of 72 patients who were clinically diagnosed with CTS were evaluated during the study period. Electrophysiological studies confirmed who met the inclusion or exclusion criteria. Out of the 72 recruited patients, three patients did not attend the follow-up meeting, while the remaining 69 patients were monitored up to four months after the procedure. Table 3 lists the demographic characteristics of the recruited participants, which were comparable in both groups with no significant differences noted between the two groups.

**Table 3:** Demographic data of the patients recruited in this study CTS: carpal tunnel syndrome

Label	Triamcinolone (n = 36)	Dexamethasone (n = 33)
Age (years)		
Mean ± standard deviation (SD)	45.22 ± 10.6	42.64 ± 10.99
Gender		
Male, n (%)	7 (19.4)	4 (12.9)
Female, n (%)	29 (80.6)	29 (87.1)
Occupation, n (%)		
Housewife	19 (52.8)	21 (63.7)
Manual worker	11 (30.5)	8 (24.2)
Others	6 (16.7)	4 (12.1)
Dominant hand, n (%)		
Right	33 (91.7)	31 (93.9)
Left	3 (8.3)	2 (6.1)
Side of symptoms, n (%)		
Right	19 (52.8)	19 (57.6)
Left	17 (47.2)	14 (42.4)
Dominant hand	18 (50)	19 (57.6)
Nondominant hand	18 (50)	14 (42.4)
Severity of CTS, n (%)		
Mild	22 (61.1)	15 (45.5)
Moderate	14 (38.9)	18 (54.5)

**Figure 2:**
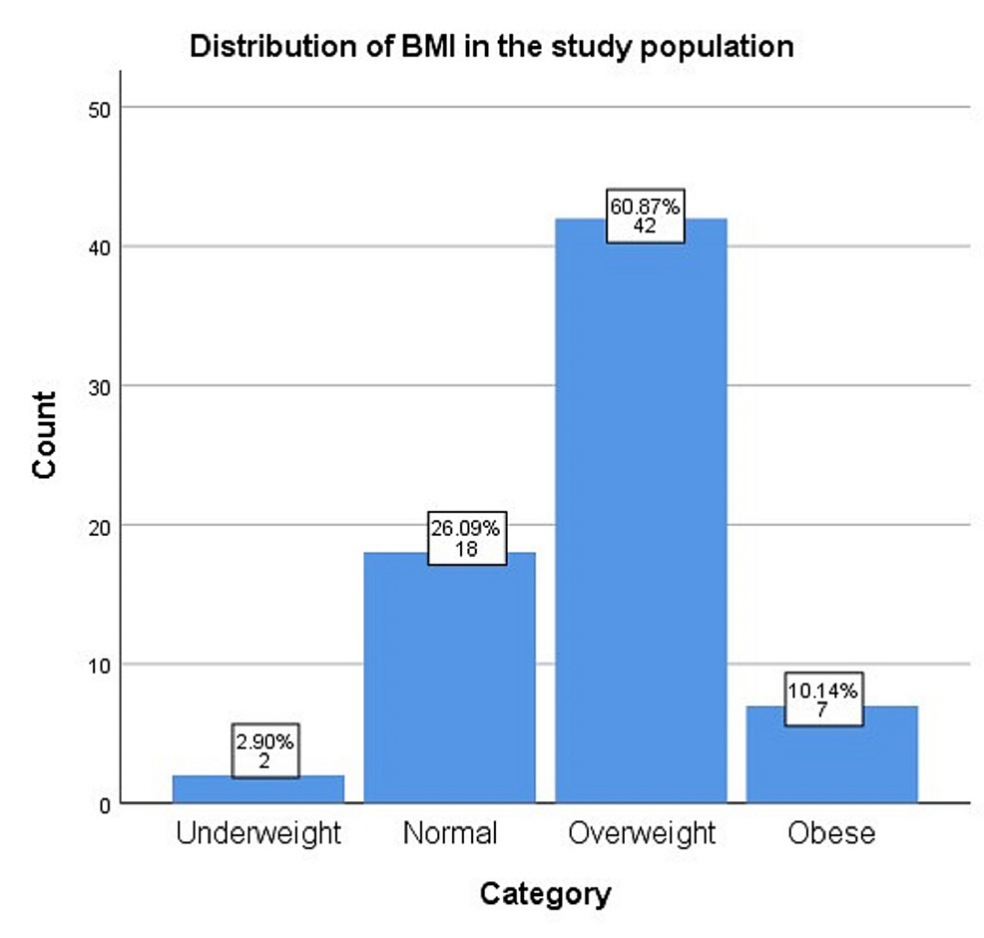
Categorization of the study population according to BMI BMI: body mass index

Figure 2 shows the distribution of the body mass index (BMI) for the studied population according to the World Health Organization cutoff categories. There were two (2.9%) underweight, 18 (26.09%) normal, 42 (60.87%) overweight, and seven (10.14%) obese women in this population. The striking majority of patients were in the overweight category.

Table 4 lists the improvement observed in each group for each of the assessed parameters. As is evident from the table, the P values associated with improvements at the second and fourth months after injection each for Phalen’s test time, VAS score, BCTQ score, and the NCS parameters (SNCV and DML) were <0.05 in both groups. Thus, it can be inferred that there were significant improvements in both groups for each of the assessed parameters.

**Table 4:** Intragroup comparison VAS: visual analog scale BCTQ: Boston carpal tunnel questionnaire SNCV: sensory nerve conduction velocity DML: distal motor latency

Parameter	Dexamethasone group (n = 33)			Triamcinolone group (n = 36)		
Phalen’s test time	Mean	Standard deviation (SD)	P value	Mean	SD	P value
At baseline	33.73	8.304		35.5	8.687	
After two months	51.45	5.154	<0.05	52.81	4.845	<0.05
After four months	42.88	3.806	<0.05	43.22	4.817	<0.05
VAS score						
At baseline	6.36	0.994		6.17	0.910	
After two months	1.85	0.870	<0.05	1.39	1.225	<0.05
After four months	2.06	1.116	<0.05	1.75	1.422	<0.05
BCTQ score						
At baseline	49.18	17.74		51.81	17.139	
After two months	21.15	3.043	<0.05	22.78	6.551	<0.05
After four months	21.61	4.723	<0.05	23.83	7.193	<0.05
SNCV						
At baseline	37.38	6.2455		39.217	6.2764	
After four months	44.394	6.7027	<0.05	45.428	6.2912	<0.05
DML						
At baseline	4.242	0.7150		4.244	1.1816	
After four months	3.367	0.5605	<0.05	3.300	1.0871	<0.05

Table 5 shows the intergroup comparison of the mean Phalen’s test time at baseline and after two and four months. The mean baseline Phalen’s test time in the dexamethasone group was 33.73 ± 8.304 seconds and that of the triamcinolone group was 35.5 ± 8.687 seconds. The P value was >0.05. Hence, there is no significant difference between the two groups at baseline with respect to Phalen’s test time. The mean Phalen’s test time at the second month in the dexamethasone group was 51.45 ± 5.154 seconds and that of the triamcinolone group was 52.81 ± 4.845 seconds. The P value was >0.05. Hence, there is no significant difference between the two groups in the follow-up at the second month. The mean Phalen’s test time at the fourth month in the case of the dexamethasone group was 42.88 ± 3.806 seconds and that of the triamcinolone group was 43.22 ± 4.817 seconds. The P value was >0.05. Hence, there is no significant difference between the two groups even four months later.

**Table 5:** Intergroup comparison of Phalen’s test time *Independent samples t-test

Phalen’s test time	Treatment	Mean	Standard deviation (SD)	P value*
At baseline	Dexamethasone	33.73	8.304	
	Triamcinolone	35.50	8.687	0.390
After two months	Dexamethasone	51.45	5.154	
	Triamcinolone	52.81	4.845	0.266
After four months	Dexamethasone	42.88	3.806	
	Triamcinolone	43.22	4.817	0.745

Table 6 lists the intergroup comparison of the mean VAS score at baseline (VAS0), after two months (VAS2), and after four months (VAS4). As is evident from the table, the P value is >0.05 at baseline and at the second and fourth months, which implies that there was no significant difference between the two groups in terms of the VAS score.

**Table 6:** Intergroup comparison of VAS score VAS: visual analog scale

VAS score	Treatment	Mean	Standard deviation (SD)	P value
At baseline	Dexamethasone	6.36	0.994	
	Triamcinolone	6.17	0.910	0.393
After two months	Dexamethasone	1.85	0.870	
	Triamcinolone	1.39	1.225	0.079
After four months	Dexamethasone	2.06	1.116	
	Triamcinolone	1.75	1.422	0.319

Table 7 shows the intergroup comparison of the mean BCTQ scores at baseline (BCTQ0) and after two months (BCTQ2) and four months (BCTQ4). As is evident from the table, the P value is >0.05 at baseline and at the second and fourth months, which implies that there was no significant difference between the two groups in terms of the BCTQ score.

**Table 7:** Intergroup comparison of BCTQ score BCTQ: Boston carpal tunnel questionnaire

BCTQ score	Treatment	Mean	Standard deviation (SD)	P value
At baseline	Dexamethasone	49.18	17.747	
	Triamcinolone	51.81	17.139	0.534
After two months	Dexamethasone	21.15	3.043	
	Triamcinolone	22.78	6.551	0.197
After four months	Dexamethasone	21.61	4.723	
	Triamcinolone	23.83	7.193	0.137

Table 8 shows the intergroup comparison of mean NCS parameters, namely, the SNCV and DML, at baseline and after two and four months. As is evident from the table, the P value is >0.05 at baseline and after the second and fourth months, which implies that there was no significant difference between the two groups in terms of the NCS parameters as well.

**Table 8:** Intergroup comparison of NCS parameters NCS: nerve conduction study SNCV: sensory nerve conduction velocity DML: distal motor latency

NCS parameter	Treatment	Mean	Standard deviation (SD)	P value
SNCV at baseline	Dexamethasone	37.380	6.2455	
	Triamcinolone	39.217	6.2764	0.228
SNCV after four months	Dexamethasone	44.394	6.7027	
	Triamcinolone	45.428	6.2912	0.511
DML at baseline	Dexamethasone	4.242	0.7150	
	Triamcinolone	4.244	1.1816	0.995
DML after four months	Dexamethasone	3.367	0.5605	
	Triamcinolone	3.300	1.0871	0.753

Table 9 shows the intergroup comparison of postprocedural pain duration, which was significantly more pronounced in the triamcinolone group (mean duration: 5.31 ± 1.037 days) compared with the dexamethasone group (mean duration: 2.67 ± 0.692 days) with a P value < 0.05.

**Table 9:** Intergroup comparison of postprocedural pain duration

Label	Mean (days)	Standard deviation (SD)	P value
Dexamethasone	2.67	0.692	
Triamcinolone	5.31	1.037	0.048

## Discussion

CTS is a very commonly encountered condition in our clinical setups. If left untreated, the initial sensory symptoms can progress to motor weakness of the hand that will cause difficulties in holding and gripping objects and will thus increase the patient’s dependence on others for their daily activities. Many patients report increased symptoms during the night hours that disturb their sleep patterns and result in daytime sleepiness, difficulties, and decreased efficiency in executing their daily living activities, thereby decreasing the quality of life.

Similar to the disease prevalence, we found that CTS was much more common in females than in males (84% versus 16%), as also reported by Mondelli et al. [2]. Obesity is a well-recognized risk factor for the development of CTS as mentioned by Genova et al. [17]. In our study, we found that 60% of the patients were found to be in the overweight category.

Our study was conducted in the western state of Rajasthan where milking of cows is a very common daily task in many households. Accordingly, this activity causes repetitive trauma to the wrist, which is a primary factor predisposing the population to the development of CTS.

Local corticosteroid injections have been successfully used for the treatment of CTS for more than half a century and have been found to be effective [11,18,19]. Different authors, such as Marshall et al. [19], Piazzini et al. [20], Peters-Veluthamaningal et al. [21], Ertem et al. [22], and others, had previously shown that local corticosteroid injections in CTS had good short-term efficacy in reducing the symptoms of CTS. In our study, we obtained similar results, which imply that steroids are indeed effective in reducing the symptoms of CTS.

To this date, there has been no specification in the literature regarding any particular corticosteroid to be used as the standard treatment in CTS. It has been noted that triamcinolone acetonide is currently one of the most commonly used steroid injections for the treatment of CTS. However, similar to the findings by Mackinnon et al. that owing to its characteristics of being water-insoluble, due to its potential to form white sediments, and property of crystallization at the injection site, triamcinolone is more prone to develop more adverse events after local injection [12]. Additionally, we made similar observations that confirmed our findings in our study as postinjection flare and injection site pain were significantly more pronounced in the triamcinolone group compared with the dexamethasone group. Mackinnon et al. had also reported that triamcinolone caused widespread axonal and myelin degeneration, and if a physician accidentally injects triamcinolone acetonide directly into the nerves, it could cause permanent nerve injury [12]. However, given that we performed all the injections under ultrasonic guidance, we did not encounter any nerve injury events in either group. Thus, the propensity of more nerve damage with triamcinolone cannot be commented on based on our study.

Furthermore, as reported by Habib et al. and Wang et al., the use of local dexamethasone injections did not have any systemic side effects, such as changing the blood sugar level of the patients, which was also confirmed in our study as the blood sugar values of the patients who received dexamethasone in our study remained in the normal range after the procedure [23,24].

Only a few studies have been conducted on the efficacy of dexamethasone for the treatment of CTS. Niempoog et al. studied the efficacy of dexamethasone injection for the treatment of CTS in pregnancy and showed that it was an effective treatment option for controlling the symptoms of CTS in pregnant women [13]. Moghtaderi et al. also reported significant improvement in pain intensity and electrophysiological parameters after dexamethasone injection in pregnant women with CTS [14]. However, owing to ethical concerns, we have not included pregnant women in our study; accordingly, they were managed conservatively without any injections.

Dilokhuttakarn et al. in a prospective, randomized, double-blind, controlled, clinical trial compared the efficacy of dexamethasone and triamcinolone injections in CTS and found that dexamethasone injections were effective and significantly improved the positive Phalen’s test time compared with triamcinolone acetonide, but there was no significant difference between the two groups [15]. Our study also yielded similar results with significant improvements in all the assessed parameters in both groups, but there were not any significant differences between the two groups in any of the assessed parameters.

Strengths

Prior to the recruitment for the study, all the clinically diagnosed cases were confirmed by electrophysiological tests.

Limitations

One limitation of the current study is the reduced sample size. In our study, the recruitment period was 18 months, during which we could recruit only 69 patients despite the fact that we had initially expected more. However, the coronavirus disease pandemic and the subsequent lockdown drastically affected the patient inflow and led to a reduction in the number of recruited patients. This was an open-label study, and no blinding was done. We excluded pregnant women with CTS from our study owing to ethical concerns. The follow-up duration was only four months after the injection. Accordingly, the long-term efficacy of these steroids could not be assessed.

## Conclusions

Our study concludes that both dexamethasone and triamcinolone injections significantly improve the symptoms and electrophysiological parameters of carpal tunnel syndrome up to four months after the injection without any significant difference between the two groups. Postprocedural pain, however, was found to be significantly more pronounced in the triamcinolone group compared with the dexamethasone group. Our findings show that it is likely that the injection of dexamethasone sodium phosphate can be used as a safe and equally effective alternative to the conventionally used injection of triamcinolone acetonide in cases of mild to moderate CTS.

This is relevant in the current practice of physiatrists and pain physicians who regularly inject steroids to relieve symptoms of carpal tunnel syndrome. Using nonparticulate steroids can make the procedure less painful for the patient. The use of ultrasound guidance is recommended as it can avoid the potential complications caused by blind injection into the nerve.

Our results may be further established with future bigger study designs. Comparison of different doses of dexamethasone and triamcinolone in CTS may be an area of future research.
